# Organic–inorganic hybrid ferrocene/AC as cathodes for wide temperature range aqueous Zn-ion supercapacitors†

**DOI:** 10.1039/d2ra02907c

**Published:** 2022-06-23

**Authors:** Shuangyu Li, Shu Zhang, Tingting Feng, Haiping Zhou, Mengqiang Wu

**Affiliations:** School of Materials and Energy, University of Electronic Science and Technology of China 2006 Xiyuan Avenue, West High-Tech Zone Chengdu 611731 China shuzhang@uestc.edu.cn mwu@uestc.edu.cn; The Yangtze Delta Region Institute (Huzhou), University of Electronic Science and Technology of China Huzhou 313001 China

## Abstract

Organic and inorganic materials have their own advantages and limitations, and new properties can be displayed in organic–inorganic hybrid materials by uniformly combining the two categories of materials at small scale. The objective of this study is to hybridize activated carbon (AC) with ferrocene to obtain a new material, ferrocene/AC, as the cathode for Zn-ion hybrid supercapacitors (ZHSCs). The optimized ferrocene/AC material owns fast charge transfer kinetics and can obtain pseudo-capacitance through redox reaction. Due to the introduction of ferrocene/AC, the ZHSCs exhibit remarkable electrochemical performances relative to that using ferrocene cathode, including high discharge specific capacity of 125.1 F g^−1^, high energy density (up to 44.8 Wh kg^−1^ at 0.1 A g^−1^) and large power density (up to 1839 W kg^−1^ at 5 A g^−1^). Meanwhile, the capacity retention rate remains 73.8% after 10 000 charge and discharge cycles. In particular, this cathode material can be used at low temperatures (up to −30 °C) with 60% capacity remained, which enlarges the application temperature range of ZHSCs. These results of this study can help understand new properties of organic–inorganic hybrid materials.

## Abstract 2

Organic and inorganic materials have their own advantages and limitations, and new properties can be displayed in organic–inorganic hybrid materials by uniformly combining the two categories of materials at small scale.

## Graphical abstract

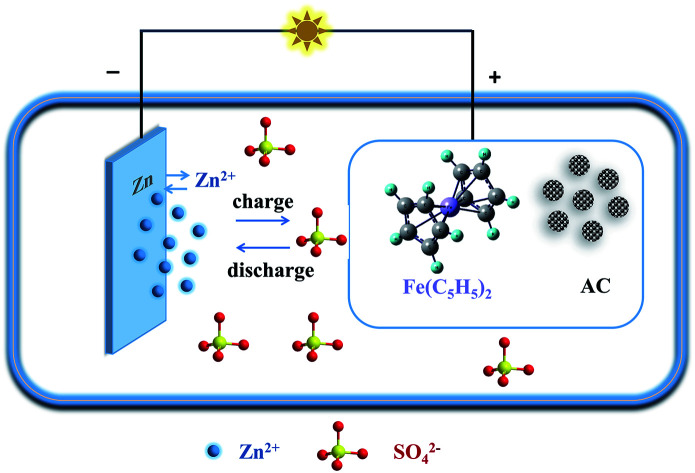

## Introduction

Zn anodes have long been used in electrochemical energy storage devices, especially in primary and secondary aqueous batteries,^1^ because of the advantages of Zn anodes, including the high theoretical capacity (820 mA h g^−1^), low redox potential (−0.76 V *vs.* the standard hydrogen electrode), low cost and rich abundance (93.84 million tons in Earth's crust), high reversibility of deposition and stripping, long service life and extreme safety.^2–5^ With economic development, rapid growth of energy consumption and limited reservation of fossil fuels call for the increasing utilization of renewable energy (such as wind and solar energies). In this context, aqueous Zn-ion hybrid supercapacitors (ZHSCs) have emerged as a promising cost-efficient technique for large scale energy storage applications.^6,7^ As an energy storage device, ZHSCs is composed of a capacitor-type cathode, a battery-type metal zinc anode, and aqueous electrolyte containing Zn^2+^.^8^ During the charging and discharging process, Zn^2+^ deposits/dissolves on the Zn anode rapidly, which ensures a high energy density, and the simultaneous reversible adsorption/desorption process of anions on the cathode material ensures an excellent power density.^9^ Besides the benefits from Zn anodes, aqueous ZHSCs also take the advantages of supercapacitors (SCs), *i.e.*, fast charge/discharge rate, high power density, good cycling stability and environmental friendliness.^10,11^

Studies of Zn-ion energy storage devices mainly focus on the development of cathode materials,^12,13^ most of which are inorganic materials. These inorganic cathodes mainly consist of transition metal oxides and Prussian blue analogs, and manganese oxides (commonly MnO_2_) and vanadium oxides (commonly V_2_O_5_) are representative transition metal oxides.^14,15^ Ma *et al.* reported the aqueous ZHSCs using V_2_O_5_ as the cathode, showing an operating voltage range of 0–2 V, with the maximum specific capacity and energy density of 57.4 mA h g^−1^ and 34.6 Wh kg^−1^, respectively.^16^ MnO_2_ nanorods were used as the cathode in ZHSCs, showing the maximum specific capacity of 54.1 mA h g^−1^ and maximum energy density of 34.8 Wh kg^−1^, and good cycling stability with 93.4% capacity retention over 5000 charge/discharge cycles.^17^ However, manganese oxides are plagued by the issues of dissolution in aqueous electrolytes and their low electrical conductivity.^18,19^ The crystal structure of vanadium oxides is unstable,^20,21^ and the capacity of the corresponding aqueous ZHSCs devices decreases rapidly during Zn^2+^ intercalation and deintercalation cycling.^22,23^

On the other hand, compared with inorganic materials, organic cathode materials have also been used in aqueous energy storage devices to couple with Zn anodes,^24,25^ and exhibited unique advantages, such as fast reaction kinetics, rich abundance of composition elements, high sustainability, facile structural design, and high capacity generated by multi-electron redox reactions.^5,26^ However, organic materials usually have the problem of inherent low electrical conductivity, which greatly increases the internal resistance, reduces the areal capacity and volume energy density.^5^ Since organic and inorganic cathode materials have their own limitations, new properties can be displayed in organic–inorganic hybrid materials by uniformly combining the two categories of materials at small scale.^27^ Xin *et al.* reported the aqueous Zn-ion hybrid energy storage device using poly(4,4′-thiodiphenol)-modified activated carbon (AC) as the cathode.^10^ The application of this hybrid cathode not only widens the voltage window from 0.2–1.8 V to 0.1–1.9 V, but also maintains the capacitance retention rate of 71% after 2000 charge–discharge cycles. Du *et al.* reported a novel organic–inorganic hybrid V_2_O_5_@polyaniline as the cathode for aqueous Zn ion batteries, showing a high specific capacitance of 61 mA h g^−1^ at 0.1 A g^−1^.^28^

In this work, ferrocene and AC are combined by a hybrid method to obtain a new organic–inorganic material ferrocene/AC. This hybridization allows fast charge transfer kinetics of the resulting cathode material, and can obtain pseudo-capacitance through redox reactions.^29,30^ Specifically, it possesses remarkable electrochemical performances, including the high discharge specific capacity of 125.1 F g^−1^, high energy density (up to 44.8 Wh kg^−1^ at 0.1 A g^−1^) and large power density (up to 1839 W kg^−1^ at 5 A g^−1^). The capacity retention rate can maintain 73.8% after 10 000 cycles. This cathode material can be used at low temperatures (up to −30 °C) with 60% capacity remained, which enlarges temperature application field of ZHSCs.

## Experimental

### Preparation of ferrocene/AC

Ferrocene was purchased from Adamas and used directly without any purification, and AC powder was obtained from Kejing (Hefei) and was sintered in argon at 480 °C for 3 h before use. These two materials were ground and mixed with a pestle and mortar in a mass ratio of 1 : 1 as the active material (denoted as ferrocene/AC). For comparison, cathodes of ferrocene, AC powder, separately, and mixtures of ferrocene and AC powder with different mass ratios other than 1 : 1 (*i.e.*, 3 : 1, 5 : 1 and 7 : 1) were also prepared using the same method. The active material, conductive additive (Super P) and polyvinylidene fluoride (PVDF) in a mass ratio of 8 : 1 : 1 were mixed, and then appropriate amount of *N*-methyl pyrrolidone (NMP) was added to form a homogeneous slurry. The resulting slurry was coated on stainless steel mesh (with a thickness of 244 μm) and dried in a vacuum oven at 70 °C for 12 h as cathode.

### Material characterizations

X-ray diffraction (XRD, 2700BH) was used to obtain the information of the diffraction pattern and the composition of materials. The scan rate was set as 5° min^−1^ in the range of 5–80°. The scanning electron microscopy (SEM, JSM-5900LV) was used to observe the microstructure and morphologies of electrode. The X-ray photoelectron spectroscopy (XPS, PHI 5000 Versa) was used to obtain the information of the chemical bonding states. The specific surface area was measured by a Brunauer–Emmett–Teller analyzer (BET, JW-BK132F). The corresponding surface areas of micropores and mesopores were calculated by *t*-plot method.

### Electrochemical characterizations

ZHSCs were assembled as CR2032 coin cells using the hybrid cathode (with ferrocene and AC in mass ratio of 1 : 1, 3 : 1, 5 : 1 and 7 : 1), Zn anode, 2 M ZnSO_4_ electrolyte and fiber glass separator. Zn foils were polished by abrasive papers before use. The electrochemical tests were performed on a CHI660E electrochemical workstation and a LAND CT2001A battery-testing instrument. The electrochemical tests include cyclic voltammetry (CV), galvanostatic charge–discharge (GCD), electrochemical impedance spectroscopy (EIS), long cycling stability, and rate performance. The voltage range of all tests were 0.2–1.8 V. The frequency of EIS was ranging from 10 mHz to 100 kHz with 5 mV AC (alternating current) amplitude.

The specific capacitance (*C*_s_, F g^−1^), energy density (*E*, Wh kg^−1^) and power density (*P*, W kg^−1^) were calculated by the following eqn (1)–(3), respectively:^31,32^1
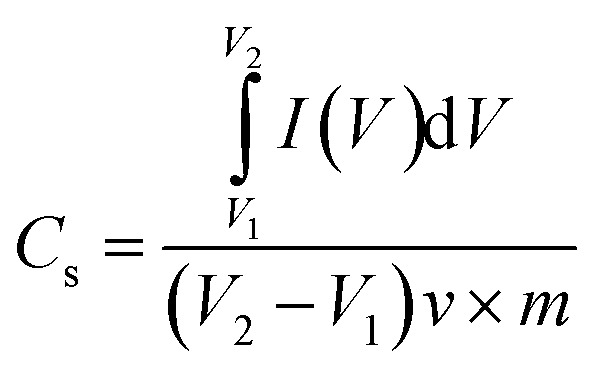
2
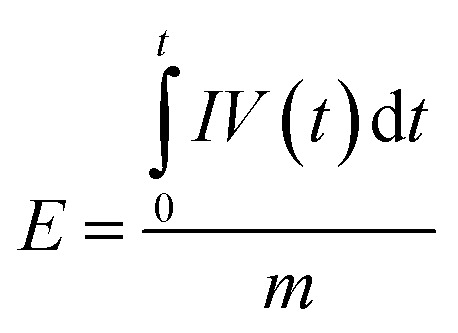
3
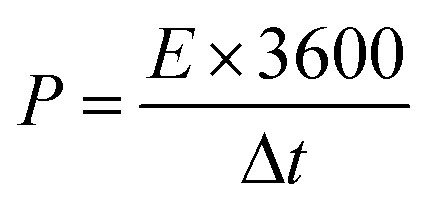
in which *I*, (*V*_2_ − *V*_1_), *v*, m and Δ*t* represent the discharge current, voltage window of the discharge process, scan rate, mass loading of the active materials based on the cathode, and discharge time, respectively. Is the area under the curve *I*/*V*.

## Results and discussion

In this study, we designed the organic–inorganic hybrid material, ferrocene/AC, as the cathode material for ZHSCs. Ferrocene was chose as the organic component, because it is a typical organo metallic molecule with rapid reversible redox rate, which has been widely used as the reference electrode in electroanalysis.^33^ The standard rate constants for ferrocene is in the range of 3.7 cm s^−1^, but it cannot be solely used as an electrode material due to its intrinsically poor electrical conductivity. For the inorganic side, the most widely used AC was chosen due to its well-defined capacitive energy storage mechanism, low cost, good conductivity and excellent stability as cathode materials for SCs. It is an effective way to improve the electrochemical performance of cathode materials in ZHSCs by introducing heteroatoms on carbon surface or redox materials.^34,35^ Due to the combination of the SCs-type cathode and battery-type anode, the ZHSCs can store charge *via* anion adsorption/desorption on the organic–inorganic hybrid materials cathode and reversible Zn^2+^ deposition/stripping on the Zn anode.^9,10,36^ Ferrocene distributed in the pores of AC provides redox active groups that can store charge.^37^

**Fig. 1:**
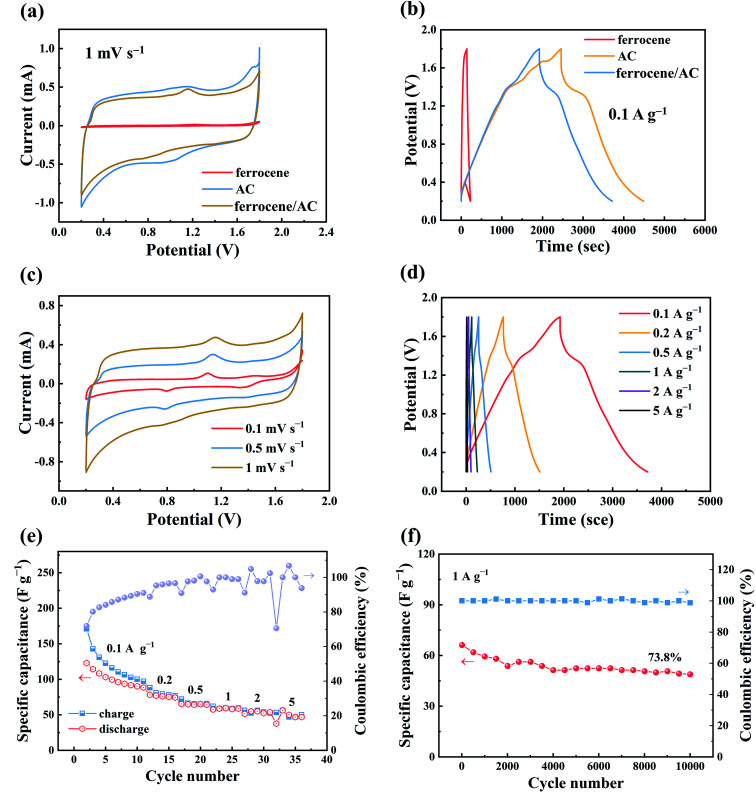
Comparison of electrochemical behaviors of ZHSCs using AC, ferrocene, and ferrocene/AC cathodes: (a) CV curves; (b) GCD profiles; electrochemical behaviors of ZHSCs using ferrocene/AC cathode: (c) CV curves; (d) GCD profiles; (e) rate performance at current densities of 0.1–5 A g^−1^; (f) long-term cycling at 1 A g^−1^.

Fig. 1a and b show the comparison of electrochemical behaviors of ZHSCs using three different cathode materials: AC, ferrocene and ferrocene/AC. Although pure ferrocene barely shows energy storage property (4.8 F g^−1^ at the scan rate of 10 mV s^−1^), in the same conditions, ferrocene/AC shows comparable specific capacitance with AC (125.1 *vs.* 107.9 F g^−1^). Given that ferrocene/AC comprises of ferrocene and AC in the mass ratio of 1 : 1, the faradaic reaction of ferrocene must contribute to the capacitance of this hybrid material. Fig. 1c shows the CV curves of ZHSCs using ferrocene/AC cathode at different scan rates, in the range of 0.1 mV s^−1^ to 1 mV s^−1^, with voltage window in between 0.2–1.8 V. All the curves show asymmetric deviation from the standard rectangular shapes of ideal SCs, suggesting that faradaic reactions take place with the hybrid material.^38^ In addition, the reduction and oxidation peaks appear at around 0.8 V and 1.1 V, respectively. As a comparison, the CV curves of pure ferrocene in Fig. S1a (in ESI)† display the oxidation and reduction peak positions at about 1.2 V and 0.7 V, respectively, showing larger polarization than ferrocene/AC material. Thus, the redox peaks in Fig. 1c can be attributed to the redox reactions of ferrocene in the charge and discharge processes,^17,39,40^ and the constant peak position without varying with the scan rate indicates the fast charge transfer kinetics in these processes. The CV curves of pure AC in Fig. S3a† is characterized by a quasi-rectangular pattern, exemplifying the typical electric double-layer behavior.^41^

**Fig. 2:**
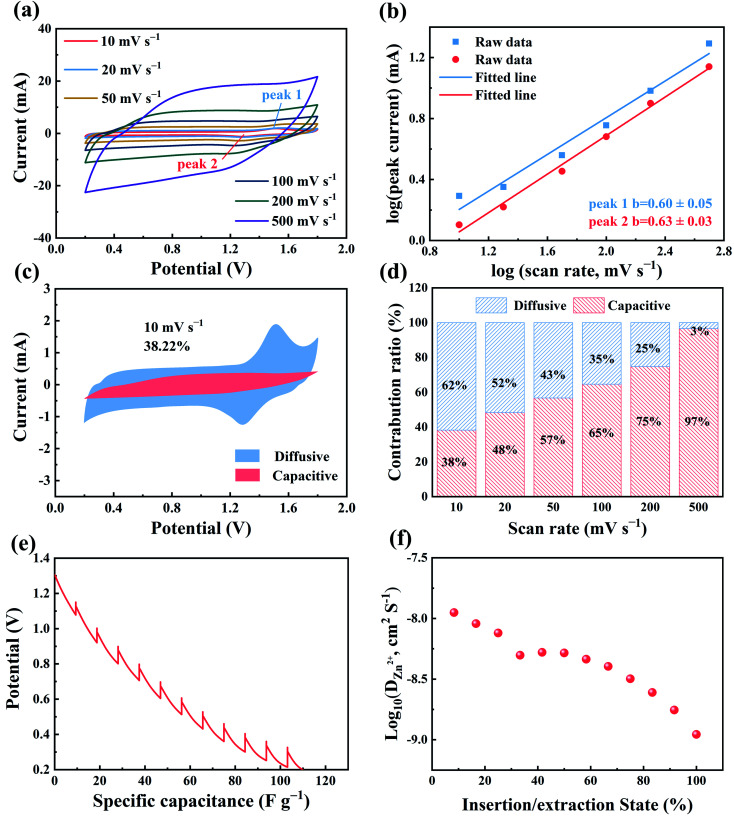
Electrochemical kinetic studies of ZHSCs using ferrocene/AC cathode. (a) CV curves; (b) fitting plot between log(*i*) and log(*v*); (c) capacitive contribution to the total stored charge from CV analysis at 10 mV s^−1^ (the red region); (d) the capacitive and diffusion contribution ratios to the total capacity at different scan rates; (e) discharge curve in GITT measurements; (f) Zn^2+^ diffusion coefficient during the discharge process.

Fig. 1d and e show good rate performance of ZHSCs using ferrocene/AC. Fig. 2a shows the discharge specific capacitance of 125.1, 108.4, 86.7, 60.8 and 33.8 F g^−1^ at the scan rates of 10, 20, 50, 100 and 200 mV s^−1^, respectively. The ZHSCs can achieve high energy and power densities up to 44.8 Wh kg^−1^ at 0.1 A g^−1^ and 1839 W kg^−1^ at 5 A g^−1^, respectively. The electrochemical performance of other samples, with different mass ratios between ferrocene and AC, were also tested using the same methods, and the results are shown in Fig. S4–S6.† The discharge specific capacitances of the control samples are 4.8 F g^−1^ for ferrocene (Fig. S1b†), 107.9 F g^−1^ for AC (Fig. S3b†), 115.4, 88.9 and 66.9 F g^−1^ for the hybrid materials with ferrocene and AC in mass ratios of 3 : 1 (Fig. S4a†), 5 : 1 (Fig. S5a†) and 7 : 1 (Fig. S6a†) at the scan rate of 10 mV s^−1^, respectively. The discharge specific capacitances of ferrocene/AC and control samples at different scan rates in the range of 10–200 mV s^−1^ are shown in Table 1.

**Table 1:** Discharge specific capacitance of hybrid materials with different mass ratios of ferrocene to AC at the scan rates of 10–200 mV s^−1^

Scan rate (mV s^−1^)	Discharge specific capacitance (F g^−1^)					
	Mass ratios of ferrocene to AC					
	1 : 0	1 : 1	3 : 1	5 : 1	7 : 1	0 : 1
10	4.8	125.1	115.4	88.9	66.9	107.9
20	2.3	108.4	98.5	70.9	52.5	90.9
50	3.3	86.7	57.7	48.1	42.9	77.3
100	/	60.8	38.5	33.7	31.3	56.8
200	/	33.8	26.9	22.3	22.1	40.9

From Table 1, it can be seen that the specific capacitances of all the hybrid materials are, more or less, higher than the sum of these for the two component materials by considering the mass fraction. For instance, at the scanning rate of 10 mV s^−1^, the discharge specific capacity (125.1 F g^−1^) of ferrocene/AC (with 1 : 1 mass ratio of ferrocene to AC) are much higher than the sum of the two component materials (4.8/2 F g^−1^ + 107.9/2 F g^−1^ = 56.4 F g^−1^). The faradaic reaction of ferrocene is responsible for the increased capacitance. Because pure ferrocene as the cathode in ZHSCs only show very small amount of capacitance, the capacitance contribution of ferrocene is probably due to the synergistic effect by combination of the two types of materials, in which AC provides pores to distribute ferrocene, and improve the charge transfer rate for the organic molecule.

To understand the electron transfer kinetics of ZHSCs using ferrocene/AC cathode, CVs were investigated at various scan rates (Fig. 2a). The peak currents (*i*, mA) of CV curve and scan rates (*v*, mV s ^−1^) have a relationship as below:

4 *i*=*av*^*b*^

where *a* and *b* are adjustable parameters, and the value of *b* expresses the charge storage mechanism: *b* close to 0.5 represents a traditional battery behavior, and energy storage in the ZHSC relies on the process of ionic diffusion control; *b* close to 1 represents a traditional pseudo-capacitor behavior; *b* in the range of 0.5–1 corresponds to devices that exhibit both battery and pseudo-capacitance properties. According to the slope of log(*i*) and log(*v*) plots for all peaks in Fig. 2b, value *b* of oxidation peak 1 and reduction peak 2 are fitted to be 0.60 ± 0.05 and 0.63 ± 0.03, respectively. The two values indicate that the charge and discharge process are controlled by both ionic diffusion and pseudo-capacitance. As shown in Fig. 2c, at scan rate of 10 mV s^−1^, 38% of the total stored charge is contributed by the capacitive-controlled process (the red region). Other capacitive contribution curves at different scan rates are shown in Fig. S7.† In Fig. 2d, the capacitive contribution ratios are 48%, 57%, 65%, 75% and 97% at different scan rates of 20, 50, 100, 200 and 500 mV s^−1^, respectively. The contribution of capacitive contribution ratio increases with the scan rate, indicating the capacitive-dominant nature and fast kinetics of ferrocene/AC at high scan rates.

Fig. 2e and f show the galvanostatic intermittent titration technique (GITT) curve of ZHSCs using the ferrocene/AC cathode in the discharge process, and the corresponding ionic diffusion coefficient (D_*Z*n_^2+^), respectively. The diffusion coefficients of Zn^2+^ are in the range of 1.11 × 10^−9^ ∼ 1.12 × 10^−8^ cm^2^ S^−1^, higher than those of 8.08 × 10^−10^ ∼ 1.07 × 10^−8^ cm^2^ S^−1^ for the AC cathode (Fig. S8†). The higher diffusion coefficients of Zn^2+^ in the former may be caused by the compatibility of organic–inorganic hybridization.^28^

Fig. 1f illustrates the specific capacitance and coulombic efficiency (CE) of ZHSCs using ferrocene/AC cathode at the current density of 1 A g^−1^. The CE retains approximately 100% after 10 000 charge and discharge cycles, and the specific capacitance remains 73.8%, displaying good cycle stability. The capacitance retention rates of samples other than ferrocene/AC are about 37% (Fig. S1d,† ferrocene), 49% (Fig. S3d,† AC), 65% (Fig. S4c,† ferrocene and AC in a mass ratio of 3 : 1), 55% (Fig. S5c,† ferrocene and AC in a mass ratio of 5 : 1) and 54% (Fig. S6c,† ferrocene and AC in a mass ratio of 7 : 1). These data show that ferrocene/AC has the lowest capacity decay after 10 000 charge and discharge cycles.

To further explore the electrochemical performance of ZHSCs using ferrocene/AC at low temperature, 2 M of ZnSO_4_ was dissolved in a modified mixing solvent.^42,43^ Owing to its relatively low freezing point (−12 °C),^44–46^ ethylene glycol (EG) was added to water as the co-solvent, in varying volume ratios of 0% (EG0) to 20% (EG20), 40% (EG40) and 60% (EG60). As shown in Fig. S9,† the ionic conductivities of these four electrolytes all decrease as the temperature decreases from 25 to −40 °C, while those of EG20, EG40 and EG60 are higher than EG0 below 0 °C. Due to the strong solvation of Zn^2+^ with EG, the hydrogen bond between EG and H_2_O is strengthened, while the hydrogen bond between H_2_O and H_2_O is weakened, leading to the higher ionic conductivity of the mixed electrolyte.^42^ EG40 can still maintain a ionic conductivity of 1.95 mS cm^−1^ at −20 °C, higher than EG20 and EG60. Hence, EG40 was chosen as the low-temperature electrolyte in this study.

**Fig. 3:**
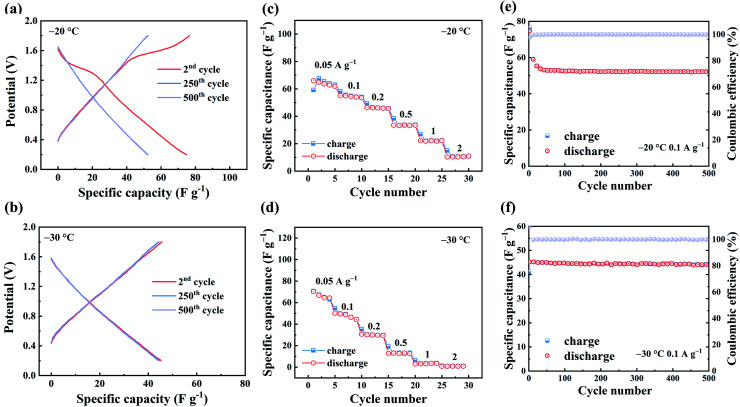
Low-temperature electrochemical performance of ZHSCs using ferrocene/AC cathode. GCD curves at 0.1 A g^−1^: (a) −20 °C; (b) −30 °C; rate performance with various current densities ranging from 0.05–2 A g^−1^: (c) −20 °C; (d) −30 °C; long-term cycling at 0.1 A g^−1^: (e) −20 °C; (f) −30 °C.

Fig. 3a and b show the GCD curves of ZHSCs using ferrocene/AC cathode at −20 °C and −30 °C, respectively. In Fig. 3a, the plot for −20 °C exhibits a plateau at 1.1–1.6 V in the 2nd cycle, which is the characteristic redox potential range of ferrocene.^47^ As the cycle progresses, there is no obvious plateau at the 25th cycle. For comparison, the GCD curves for −30 °C does not show the plateau of redox reaction of ferrocene at all cycles, and the curves at the 2nd, 250th and 500th cycles overlap well, suggesting the long-term stability of the capacitance. Fig. 3c and d show the rate performance of ZHSCs using ferrocene/AC cathode, in which the maximum discharge specific capacitance is 67 F g^−1^ at −20 °C and 70 F g^−1^ at −30 °C. The chelation between Zn^2+^ and EG weakens the solvation interaction of Zn^2+^ with H_2_O, and also enhances the hydrogen bonding between EG and H_2_O. EG40 shows a high Zn^2+^ conductivity and reversibility for energy storage, even at temperature as low as −30 °C, and thereby enables ZHSCs to operate well in a wide range of temperatures from 25 °C to −30 °C.^42,43^ Fig. S11† shows the rate performance of ZHSCs using AC cathode at low temperatures, and the maximum discharge specific capacitances are 74 F g^−1^ and 62 F g^−1^ at −20 °C and −30 °C, respectively.

Fig. 3e and f illustrate the long-term cycling of ZHSCs using ferrocene/AC cathode at the current density of 0.1 A g^−1^. Both curves show CE remains 100% after 500 cycles, reflecting the extremely cycling durability. These results confirm the excellent electrochemical performance of ZHSCs using ferrocene/AC cathode, at low temperature up to −30 °C. The rate performance of ZHSCs using ferrocene/AC cathode at −40 °C is shown in Fig. S10.† The device barely shows any capacitance at 0.2 A g^−1^, probably due to the low ionic conductivity of EG40 at −40 °C.

**Fig. 4:**
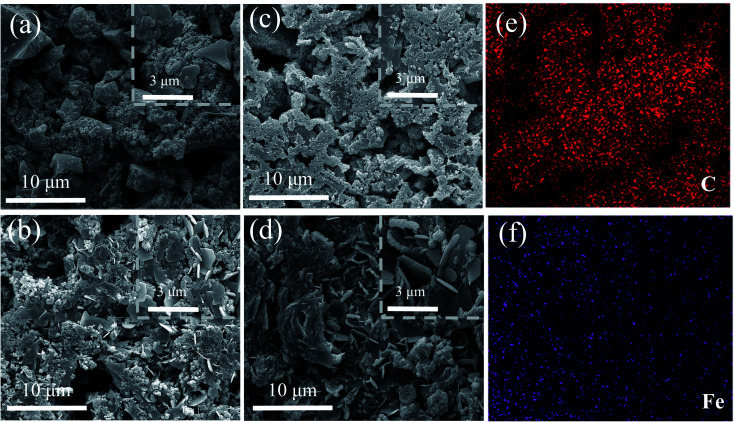
SEM images of AC cathodes (a) before and (b) after 10 000 cycles; SEM images and elements mapping of ferrocene/AC cathode (c) before and (d) after 10 000 cycles, (e) C and (f) Fe.

The SEM images in Fig. 4 show the morphology evolution of the electrodes before and after 10 000 cycles. The AC material changes from irregular shape before cycling (Fig. 4a) to that with agglomerated nanoparticles with sheet-like structure after 10 000 cycles (Fig. 4b). On contrast, morphologic change of the ferrocene/AC cathode before and after cycling is not obvious: on the agglomerated nanoparticles before cycling (Fig. 4c),^39^ sheet-like structures are evolved after cycling (Fig. 4d), which indicates that the material possesses a stable structure during cycling. This is likely to be an important reason for its excellent electrochemical performance. The lamellar spacing in ferrocene/AC cathode in Fig. 4d is obviously larger than AC cathode in Fig. 4b, which relieves the volume expansion during the charge and discharge process. Hence, the stable porous structure is the key to better rate performance and cycling stability of ZHSCs using ferrocene/AC. Fig. 4e and f show uniform distributions of C and Fe elements. The Zn anode of Zn//AC SCs after 10 000 cycles shows agglomerated particles (Fig. S12a†), which may be ZnO particles that are responsible for increasing electrode resistance and cause the failure in the charge and discharge process.^39^ The Zn anode in Zn//ferrocene/AC SCs after cycling (Fig. S12b†) shows flake-like morphology without Zn dendrite formation, indicative of uniform Zn deposition/stripping that may lead to better electrochemical performance of corresponding ZHSCs.^39^

**Fig. 5:**
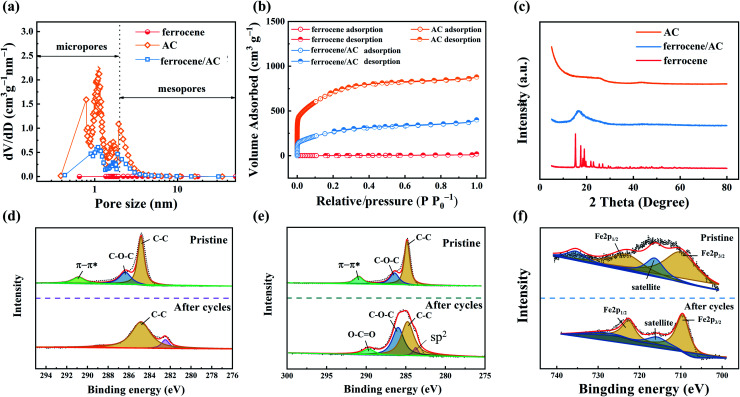
(a) Pore size distribution curve; (b) N_2_ adsorption/desorption isotherms; (c) XRD patterns of AC, ferrocene and ferrocene/AC; XPS images of (d) C 1s spectrum of AC cathode; (e) C 1s spectrum of ferrocene/AC cathode; (f) Fe 2p spectrum of ferrocene/AC cathode.

Fig. 5a shows the pore size distribution curves of AC, ferrocene and ferrocene/AC. Among these materials, micropores with a pore size of 0.8–2 nm dominate the pore structures of AC and ferrocene/AC, while in ferrocene no obvious micropores were found. In Fig. 5b, both AC and ferrocene/AC can be divided into the first-type adsorption isotherm curve, while ferrocene can be divided into the fourth-type adsorption isotherm curve. These curves all rise rapidly when the relative pressure is less than 0.01, because the adsorption occurs in micropores and small mesopores.^48,49^ As shown in Table S1,† the specific surface areas of ferrocene, ferrocene/AC and AC is 4.8, 961.6 and 2458.6 m^2^ g^−1^, respectively. The double layer capacitance of AC is retained in ferrocene/AC, and ferrocene is introduced to increase the redox capacitance. Therefore, such hybrid compound is favorable for electrochemical energy storage in ZHSCs.

The crystalline phases of AC, ferrocene and ferrocene/AC are discriminated by XRD in Fig. 5c. The XRD pattern of ferrocene is characterized by distinct peaks derived from the bulk crystalline structure. AC shows a broad peak of carbon (101) diffraction at 44° and carbon (002) diffraction at 26°. Meanwhile, a broad peak is observed at about 16° in the XRD pattern of ferrocene/AC. These results indicate that organic ferrocene molecules are well dispersed as nanocrystalline on the large surface of inorganic AC.^29^

The chemical composite of AC and ferrocene/AC cathode is analyzed by XPS. The C 1s spectrum of AC cathode before cycling shows three strong peaks at 284.8, 286.6 and 291.7 eV that can be assigned to the binding energy of C–C, C–O–C, and π–π*, respectively.^50^ After cycling, the C–C peak appears at 284.8 eV,^10,51^ and the lowest energy peak of 282.4 eV is attributed to environmental contamination.^52^Fig. 5e shows C 1s spectrum of ferrocene/AC cathode before and after cycling. These three peaks are the same as these of AC cathode before cycling, and after cycling, there are four peaks appear at 284.8, 286.6, 289.8 and 284.1 eV, corresponding to C–C, C–O–C, O–C=O and sp^2^-C respectively. Apparently, more oxygen-containing functional groups have been formed on ferrocene/AC cathode after cycling, which may provide additional pseudo-capacitance through faradaic reactions and improve the electrochemical performances of ZHSCs.^53–55^ This phenomenon indicates the nature of synergistic effect of the ferrocene/AC hybrid material.

Fig. 5f shows the Fe 2p spectrum of ferrocene/AC cathode before and after cycling. Before cycling, the binding energies of Fe 2p_3/2_ and Fe 2p_1/2_ are 710.4 eV and 723.5 eV, respectively. The satellite peak obtains at about 716.5 eV,^56^ with about 6.1 eV difference from the Fe 2p_3/2_ peak. Another satellite peak appearing at 735.5 eV might be the satellite peak for Fe 2p_1/2_. The binding energies of Fe 2p_3/2_, Fe 2p_1/2_ and satellite peaks after cycling, at 709.6, 722.6 eV and 715.9 eV, respectively, are all very close to those before cycling, indicating the electrochemical cycling process is reversible for ferrocene.

## Conclusion

In summary, we developed a ferrocene/AC hybrid cathode material for aqueous ZHSCs, showing high safety, wide operation temperature and long cycle life. This cathode material combines the advantages of inorganic and organic materials, in which a rapid charge transfer of ferrocene undergoes at the interface on the carbon surface of AC. Meanwhile, the large contact area between the finely dispersed ferrocene molecules and AC surface enables fast redox reactions of ferrocene, resulting in high power densities. Namely, AC matric provides abundant electroactive sites for insulate ferrocene organic material, which inherently has limited active sites for redox reaction. The ZHSCs using ferrocene/AC cathodes possess remarkably improved electrochemical performance related to that using ferrocene, including the high discharge specific capacitance of 125.1 F g^−1^, high energy density up to 44.8 Wh kg^−1^ at 0.1 A g^−1^, and large power density up to 1839 W kg^−1^ at 5 A g^−1^. The capacity retention rate remains 73.8% after 10 000 charge and discharge cycles. In particular, this cathode material can be used at low temperatures (up to −30 °C) with 60% capacity remained, which enlarges the application temperature range of ZHSCs. The findings of this study can help explore new application of organic–inorganic hybrid materials in the field of energy storage materials.

## Conflicts of interest

There are no conflicts to declare.

## Supplementary Material

RA-012-D2RA02907C-s001
