# Supplementary material for: Organic–inorganic hybrid ferrocene/AC as cathodes for wide temperature range aqueous Zn-ion supercapacitors
Source: RSC Adv. 2022 Jun 23;12(29):18466–74. doi: 10.1039/d2ra02907c (PMC9218876; doi:10.1039/d2ra02907c)
Supplement: RA-012-D2RA02907C-s001 [file RA-012-D2RA02907C-s001.pdf]

Electronic Supplementary Material (ESI) for RSC Advances.
This journal is © The Royal Society of Chemistry 2022

Organic-inorganic hybrid ferrocene/AC as cathodes for wide temperature range aqueous Zn-ion supercapacitors

Shuangyu Li,[a] Shu Zhang,*[ab] Tingting Feng,[ab] Haiping Zhou,[ab] and Mengqiang Wu*[ab]

[a]School of Materials and Energy, University of Electronic Science and Technology of China, 2006 Xiyuan Avenue, West High-Tech Zone, Chengdu 611731, China

[b]The Yangtze Delta Region Institute (Huzhou), University of Electronic Science and Technology of China, Huzhou 313001, China

Corresponding Authors

*Email: shuzhang@uestc.edu.cn (S.Z.)

*Email: mwu@uestc.edu.cn (M.W.)

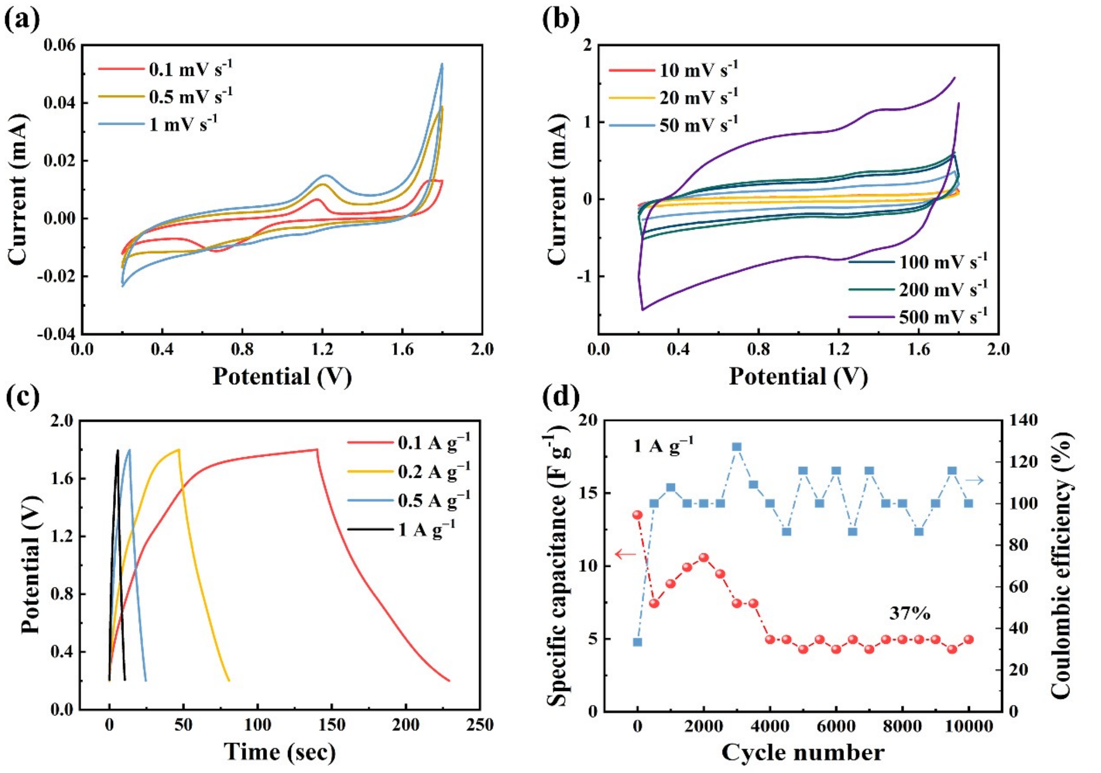

Fig. S1 Electrochemical behaviors of ZHSCs using ferrocene cathode (a) CV curves of low scan rates (0.1–1 mV s^{-1}); (b) CV curves of high scan rates (10–500 mV s^{-1}); (c) GCD profiles; (d) Long-term cycling at 1 A g^{-1}.

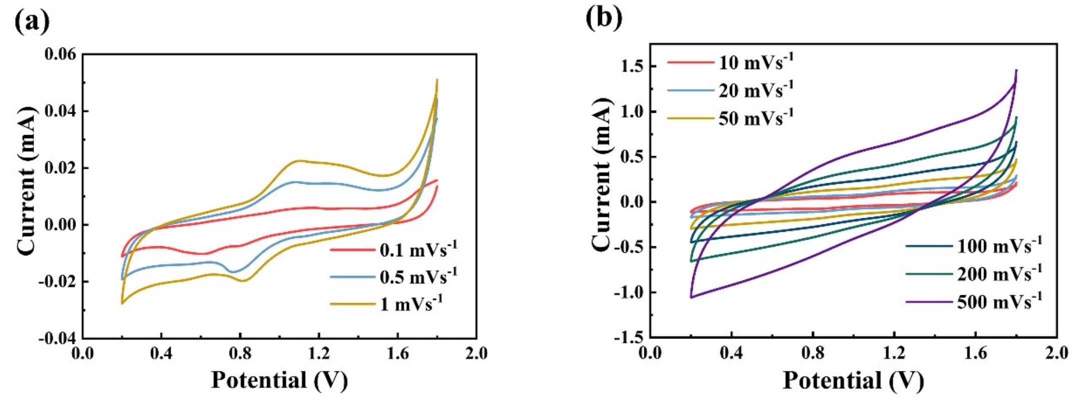

Fig. S2 CV curves of ZHSCs using ferrocene cathode in 1 M $ZnSO_4$ (a) 0.1–1 mV s^{-1}; (b) 10–500 mV s^{-1}.

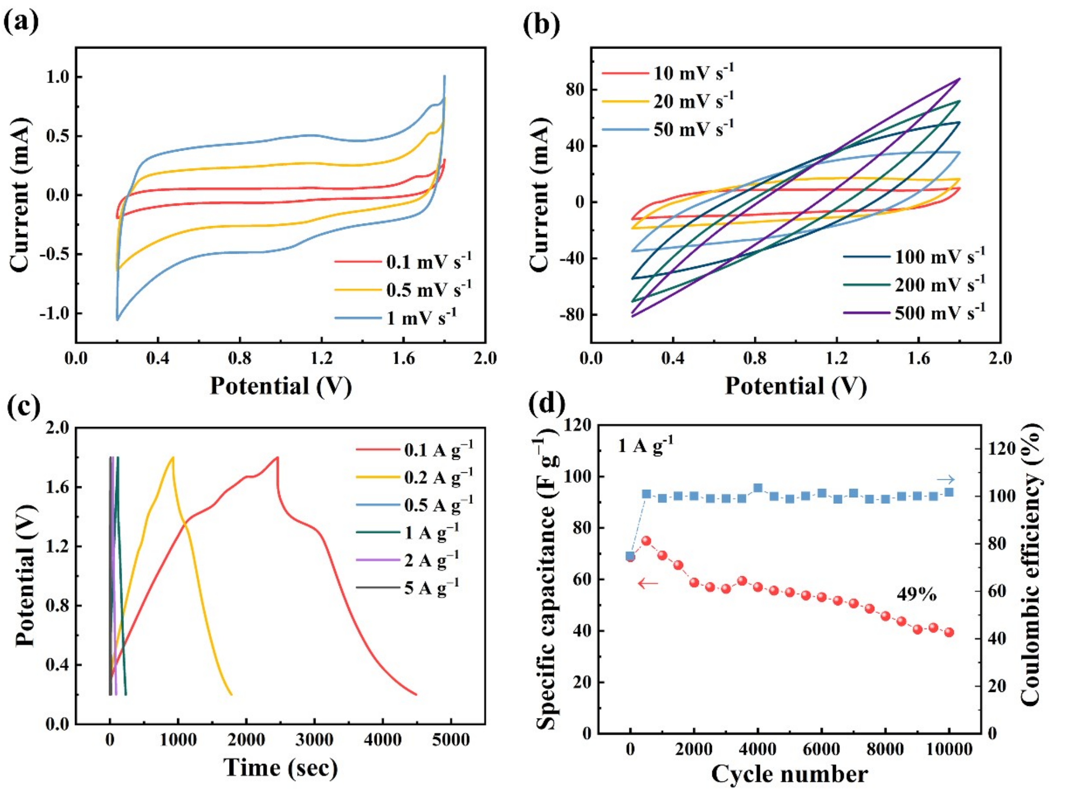

Fig. S3 Electrochemical behaviors of ZHSCs using AC cathode (a) CV curves of low scan rates (0.1–1 mV s^{-1}); (b) CV curves of high scan rates (10–500 mV s^{-1}); (c) GCD profiles; (d) Long-term cycling at 1 A g^{-1}.

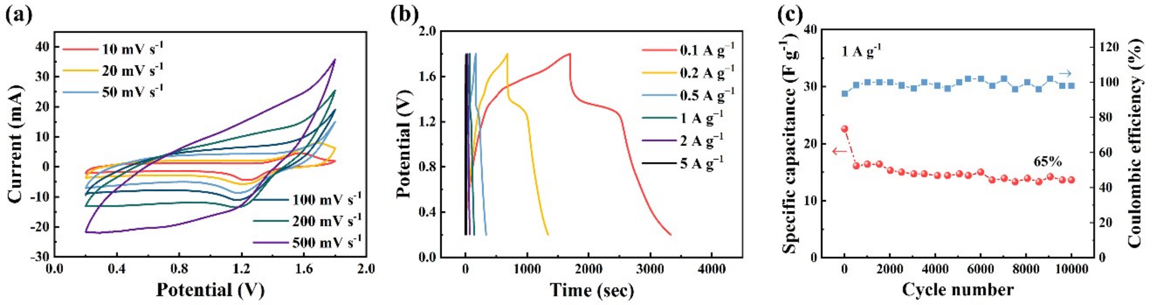

Fig. S4 Electrochemical behaviors of ZHSCs using hybrid cathode (ferrocene and AC in a mass ratio of 3:1) (a) CV curves; (b) GCD profiles; (c) Long-term cycling at 1 A g^{-1}.

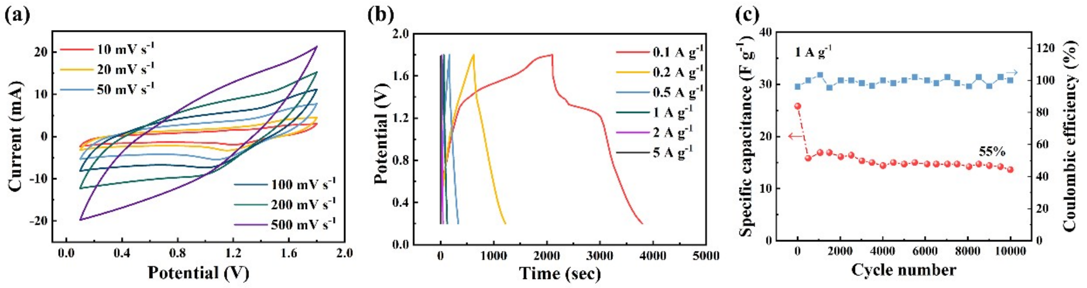

Fig. S5 Electrochemical behaviors of ZHSCs using hybrid cathode (ferrocene and AC in a mass ratio of 5:1) (a) CV curves; (b) GCD profiles; (c) Long-term cycling at 1 A g^{-1}.

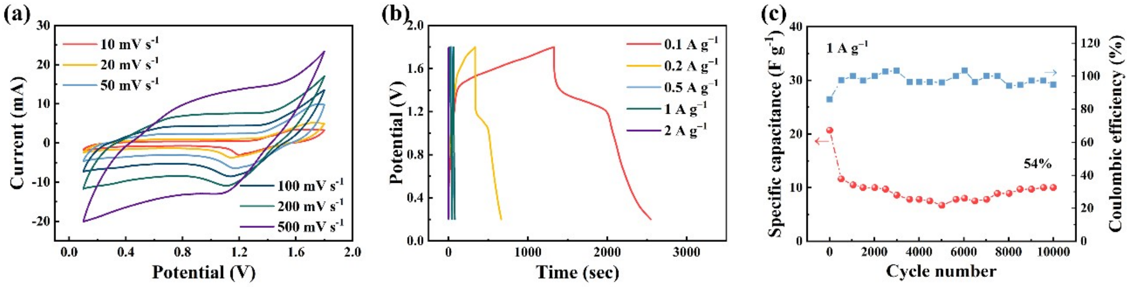

Fig. S6 Electrochemical behaviors of ZHSCs using hybrid cathode (ferrocene and AC in a mass ratio of 7:1) (a) CV curves; (b) GCD profiles; (c) Long-term cycling at 1 A g^{-1}.

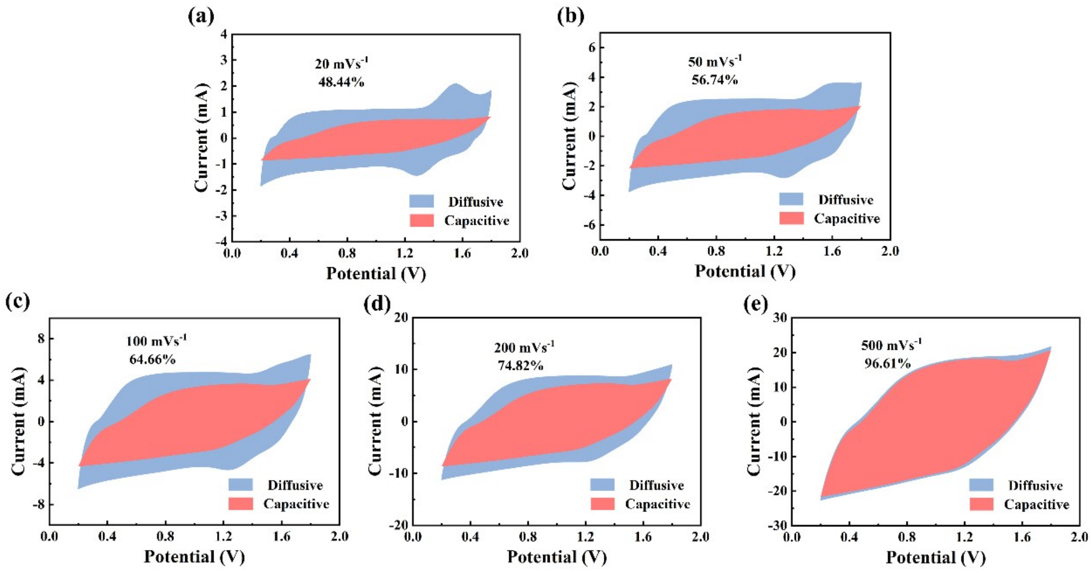

Fig. S7 Capacitive contribution to the total current from CV analysis at different scan rates (the red region).

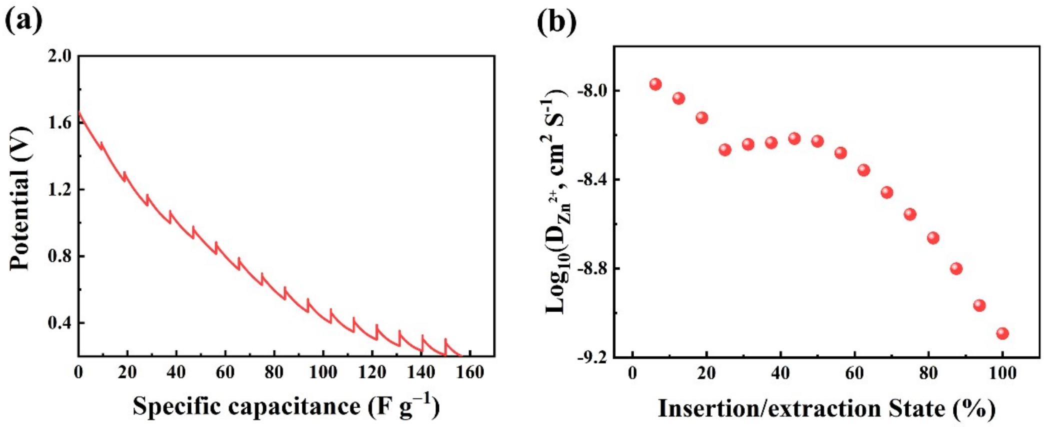

Fig. S8 (a) Discharge curve in GITT measurements of ZHSCs using AC cathode; (f) The corresponding Zn^{2+} diffusion coefficient during the discharge process.

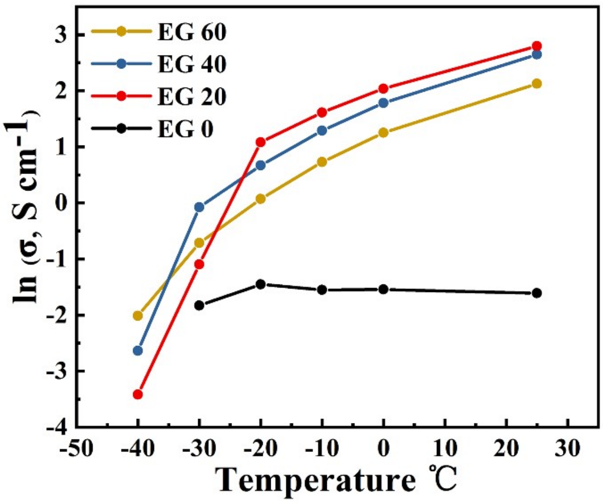

Fig. S9 The ionic conductivities of EG0, EG20, EG40 and EG60.

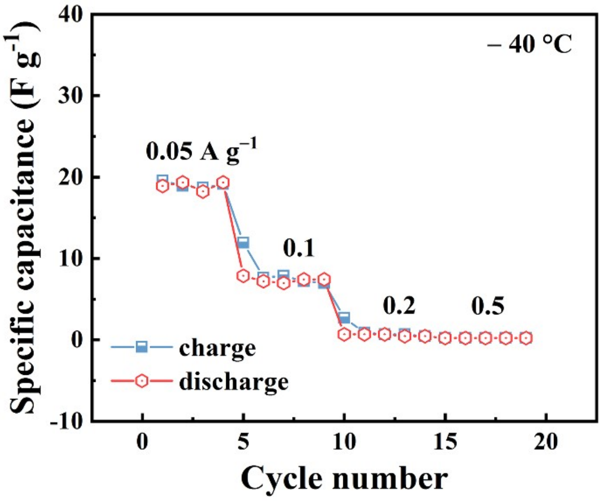

Fig. S10 Rate performance of ZHSCs using ferrocene/AC cathode at –40 °C.

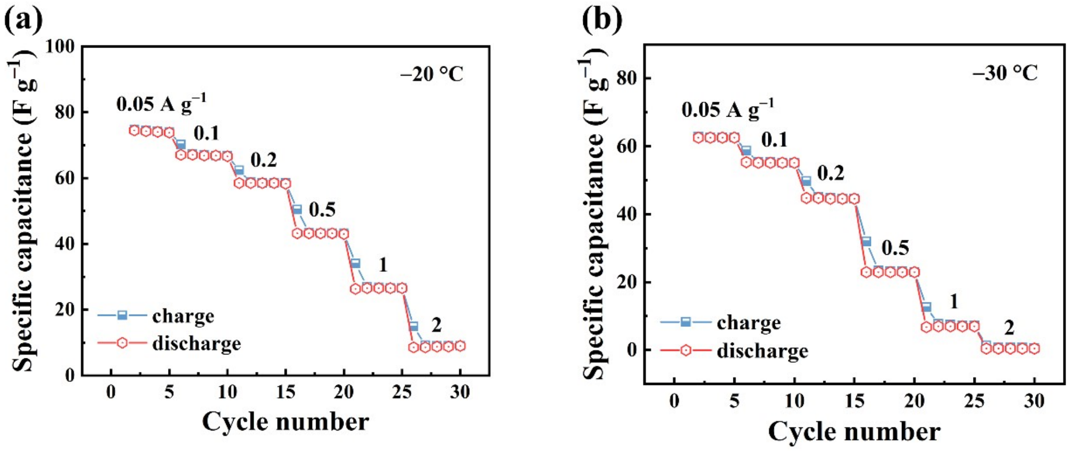

Fig. S11 Rate performance of ZHSCs using AC cathode at –20 °C and –30 °C.

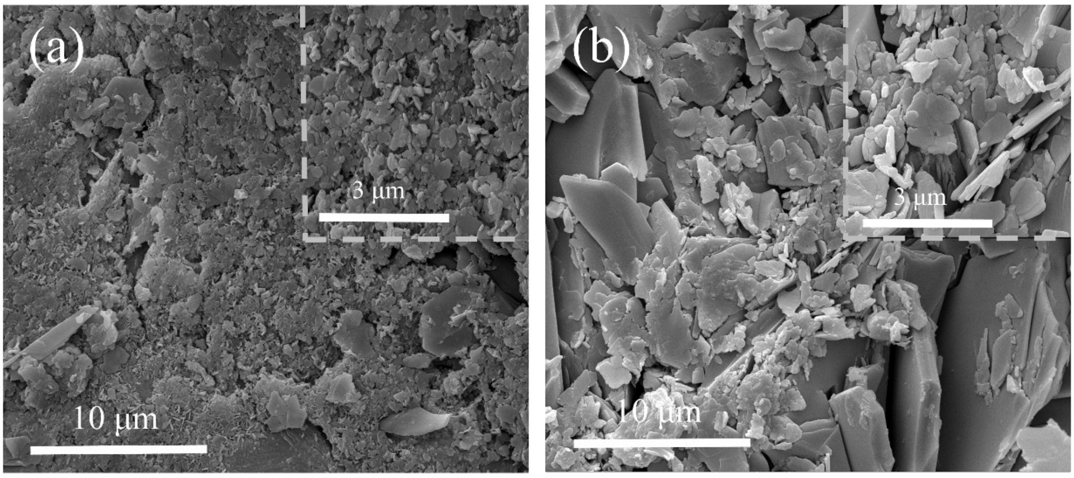

Fig. S12 SEM images after cycling (a) Zn anode in Zn // AC SCs; (b) Zn anode in Zn // ferrocene/AC SCs.

Table S1 The specific surface area, total pore volume for adsorption, total pore volume of micropores of the three samples.

Sample	Specific surface area (m^2 g^{-1})	Total pore volume for adsorption (cm^3 g^{-1})	Total pore volume of micropores (cm^3 g^{-1})
ferrocene	4.859	0.031	0.001
AC	2458.658	1.361	0.982
ferrocene/AC	961.601	0.620	0.343
